# The Association Between Late Gadolinium Enhancement by Cardiac Magnetic Resonance and Ventricular Arrhythmia in Patients With Mitral Valve Prolapse: A Systematic Review and Meta‐Analysis

**DOI:** 10.1002/clc.24316

**Published:** 2024-07-03

**Authors:** Xiaofu Tang, Weiguo Fan

**Affiliations:** ^1^ The Second Affiliated Hospital, Jiangxi Medical College Nanchang University Nanchang Jiangxi China; ^2^ Department of Cardiovascular Medicine, The Second Affiliated Hospital, Jiangxi Medical College Nanchang University Nanchang Jiangxi China

**Keywords:** cardiac magnetic resonance, late gadolinium enhancement, mitral valve prolapse, sudden cardiac death, ventricular arrhythmia

## Abstract

**Introduction:**

Malignant ventricular arrhythmia (VA) and sudden cardiac death (SCD) have been reported in patients with mitral valve prolapse (MVP); however, effective risk stratification methods are still lacking. Myocardial fibrosis is thought to play an important role in the development of VA; however, observational studies have produced contradictory findings regarding the relationship between VA and late gadolinium enhancement (LGE) in MVP patients. The aim of this meta‐analysis and systematic review of observational studies was to investigate the association between left ventricular LGE and VA in patients with MVP.

**Methods:**

We searched the PubMed, Embase, and Web of Science databases from 1993 to 2023 to identify case–control, cross‐sectional, and cohort studies that compared the incidence of VA in patients with MVP who had left ventricular LGE and those without left ventricular LGE.

**Results:**

A total of 1464 subjects with MVP from 12 observational studies met the eligibility criteria. Among them, VA episodes were reported in 221 individuals (15.1%). Meta‐analysis demonstrated that the presence of left ventricular LGE was significantly associated with an increased risk of VA (pooled risk ratio 2.96, 95% CI: 2.26−3.88, *p* for heterogeneity = 0.07, *I* ^2^ = 40%). However, a meta‐regression analysis of the prevalence of mitral regurgitation (MR) showed that the severity of MR did not significantly affect the association between the occurrence of LGE and VA (*p* = 0.079).

**Conclusion:**

The detection of LGE could be helpful for stratifying the risk of VA in patients with MVP.

## Graphical abstract

Severe ventricular arrhythmia (VA) has been reported in patients with mitral valve prolapse; this meta‐analysis indicated that the detection of late gadolinium enhancement could be helpful for stratifying the risk of VA in patients with MVP.

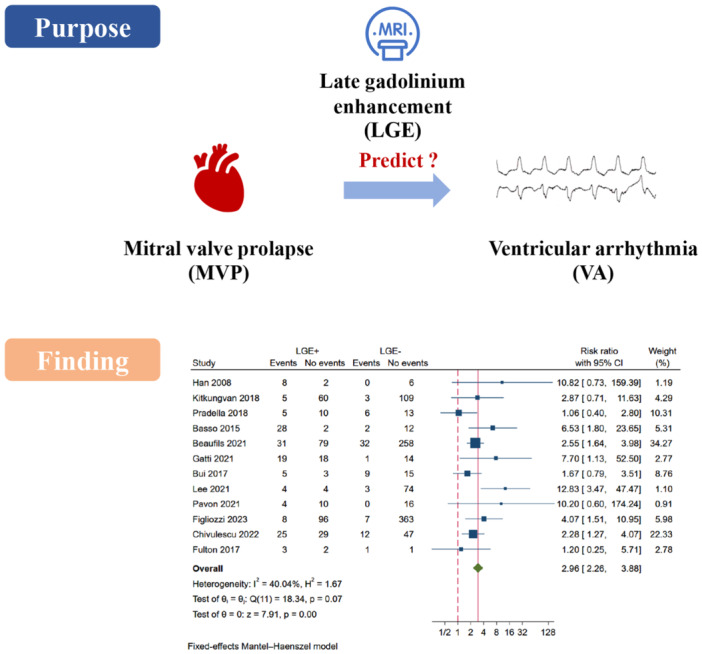

## 1 Introduction

Mitral valve prolapse (MVP) is a common valvular disease that affects approximately 2%−3% of the general population. It is characterized by displacement of the mitral valve leaflets > 2 mm into the left atrium in the sagittal view of the mitral valve [1]. Although MVP is generally considered nonfatal, complications can occur, including mitral regurgitation (MR), atrial fibrillation (AF), and heart failure [2]. Echocardiography is the preferred diagnostic method for MVP, but the American Heart Association (AHA) guidelines recommend the use of cardiac magnetic resonance (CMR) when the quality of ultrasound images cannot be properly assessed [3].

CMR is an imaging technology that provides multiple planes of view and reproducible quantitative information on the anatomy and function of the heart [4]. In recent years, the use of CMR in the evaluation of valvular heart disease has increased. This method allows for precise assessment of various parameters. These include the volume of the ventricle, the systolic function, the shape of the valve, and the degree of MR. More importantly, this technique can characterize the tissue of the myocardium and the presence of myocardial fibrosis (MF). From a pathophysiological point of view, excessive activity of the mitral valve leaflets may cause mechanical stretching of the subbasal wall and papillary muscles, resulting in fibrosis of the left ventricle. Late gadolinium enhancement (LGE) can be detected by CMR and is usually associated with the papillary muscle and the subbasal segment of the left ventricle adjacent to the prolapsed mitral valvulae [5]. The development of malignant ventricular arrhythmia (VA) seems to be related to endocardium stimulation with extended tendon [6].

One prospective cohort study estimated that the incidence of sudden cardiac death (SCD) ranged from 0.2% to 0.4% per year [7]. Due to the relatively high prevalence of MVP in the general population, it is essential to identify patients at risk for arrhythmic death.

While several recent studies have evaluated the potential association between LGE and VA in patients with MVP, other studies have found no association [2, 5, 8]. Thus, the role of the CMR in predicting VA among patients with MVP patients remains uncertain. The objective of the present study was to conduct a meta‐analysis to examine whether the presence of LGE is associated with VA among MVP patients who underwent CMR and to determine the potential benefits of using CMR among patients with MVP.

## 2 Materials and Methods

### 2.1 Search Strategy and Selection Criteria

This systematic review and meta‐analysis was reported in accordance with the Preferred Reporting Items for Systematic Reviews and Meta‐Analyses (PRISMA) Statement and was registered in Systematic Reviews (CRD42023406970).

We selected Embase, PubMed, and Web of Science from 1993 to 2023 to identify relevant case‒control studies, cross‐sectional studies, and cohort studies. The following text words and Medical Subject Heading terms were used in the search: all spellings of “MVP,” “Magnetic Resonance Imaging,” “Arrhythmias, Cardiac,” “Death, Sudden, Cardiac,” “CMR,” and “LGE” (Table S1).

### 2.2 Study Selection and Data Extraction

Studies that compared the incidence of VA between MVP patients with and without LGE eligible. The exclusion criteria were as follows: reviews, case reports, studies without specific data, and studies without assessments of gadolinium enhancement and arrhythmic events.

The investigators initially reviewed the study titles, article types, and abstracts. After the initial screening, the researcher read the full text to obtain detailed information. The researchers independently screened the studies according to the inclusion and exclusion criteria.

The following data were extracted from the eligible trials: sample size, sex, age, LV ejection fraction, AF, MR, VA, and presence of LGE by CMR. The investigators assessed bias according to the PRISMA recommendations. The Newcastle‒Ottawa Scale was used to assess the quality of cohort and case‒control studies, and the Joanna Briggs Institute scale was used to assess the quality of cross‐sectional studies.

The literature selection, data extraction, and quality evaluation processes were independently conducted by two researchers.

### 2.3 Statistical Analysis

We evaluated the impact of CMR gadolinium enhancement on VA in patients with MVP. The relative risk (RR) of the individual studies and the 95% confidence intervals (CIs) were calculated on the basis of the number of events extracted from each study before the pooling of the data. The Cochran *Q* test and *I*² test were used to assess between‐study heterogeneity. A *p* value of the Cochran *Q* test less than 0.05 or an I [2] greater than 50% indicated significant heterogeneity between studies, and in such cases, a random effects model was subsequently used to obtain pooled estimates of RRs. Otherwise, a fixed effects model was used for pooled analyses. The presence of publication bias was analyzed using funnel plots, and Begg's and Egger's tests were used to determine the significance of publication bias (defined as *p* < 0.1). We used Review Manager and Stata 17.0 for all the statistical analyses. In addition, we performed a meta‐regression analysis based on the proportion of patients with moderate to severe MR reported in each study. Finally, we excluded studies with fewer than 20 participants. We also performed a sensitivity analysis using the leave‐one‐out method to verify the robustness of the results.

## 3 Results

### 3.1 Study Selection

**Figure 1:**
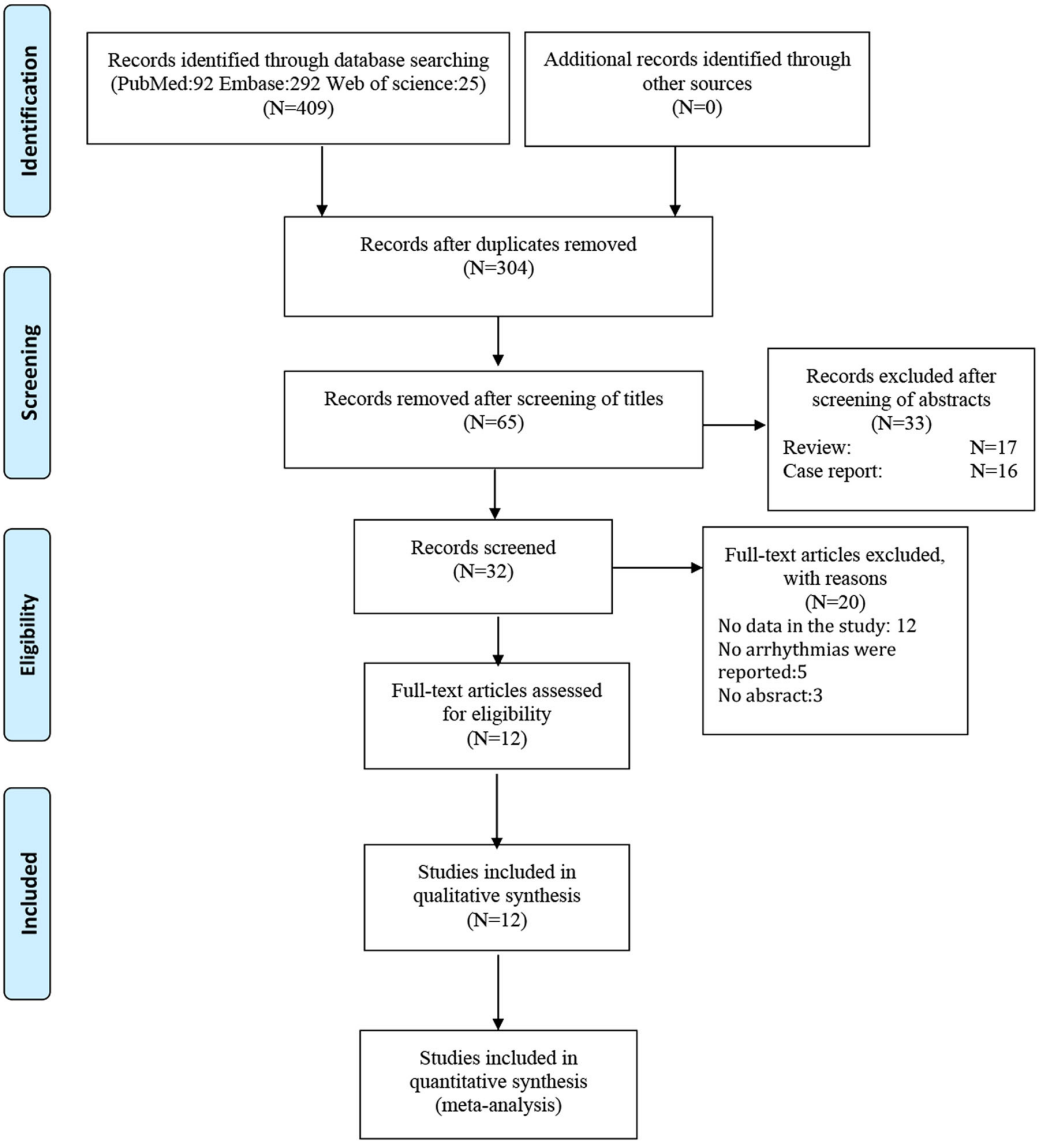
A flowchart depicting the literature screening process.

A flowchart depicting the literature screening process is shown in Figure 1. After removing duplicates and irrelevant articles, 65 studies were screened. Ultimately, 12 studies (with data for 1464 participants), including three case‒control studies [5, 9, 10], six cross‐sectional studies [1, 2, 4, 6, 11, 12], and three cohort studies [13, 14, 15], were eligible. Among the 53 excluded studies, 16 were case reports, 17 were reviews, 12 lacked necessary data for meta‐analysis, five did not report any arrhythmias, and three lacked full text.

### 3.2 Study Characteristics

The 12 included studies were published between 2008 and 2023 across six different countries (Italy, Switzerland, Korea, USA, Norway, and France). The characteristics of the included studies are shown in Table 1. Among the eligible studies, six reported the degree of moderate or severe MR, seven studies reported the left ventricular ejection fraction, and six studies reported the median follow‐up time. Two studies reported mitral annular disjunction. Additionally, most nonlongitudinal studies used Grade III or higher by the Lown and Wolf class as the definition of complex VA, including paired, multifocal, or R on T ventricular premature contractions. Three cohort studies reported the outcomes of sudden cardiac arrest (SCA) or SCD and sustained ventricular tachycardia (SVT) [13, 14, 15].

**Table 1:** Characteristics of studies included in meta‐analysis.

Author	Year	Study type	Sample size LGE+/LGE−	Age (years)	Male (%)	Moderate and severe MR (%)	LVEF (%)	Median follow‐up time	Mitral annular disjunction (%)	VA definition
Han	2008	Cross section	10/6	50.9 ± 12	60	82.86	65.6 ± 12	5 years	NA	Grade III or higher by Lown and Wolf class
Kitkungvan	2018	Cohort	65/112	61.1 ± 14.7	62.2	100.00	66.9 ± 8.4	3.7 years	NA	SCD, aborted SCA, and symptomatic ventricular tachycardia or fibrillation
Pradella	2018	Cross section	15/19	56 ± 14	59	64	61 ± 5	1 year	NA	Grade III or higher by Lown and Wolf class
Basso	2015	Cross section	30/14	43	34.90	NA	63	NA	NA	VF and VT, either sustained or non‐sustained
Beaufils	2021	Cohort	110/290	53 ± 15	55	71	61 ± 7	8.4 month	NA	Non‐sustained VT or life‐threatening VA history
Gatti	2021	Cross section	37/15	NA	28.85	NA	NA	NA	NA	VF and VT, either sustained or non‐sustained
Bui	2017	Cross section	8/24	NA	68.75	NA	NA	6 month	NA	Grade III or higher by Lown and Wolf class
Lee	2021	Cohort	8/77	54	54.10	84.60	60	7.2 years	13	SCA, sustained or non‐sustained VT
Pavon	2021	Case–control	14/16	50 ± 17	60	50	NA	NA	NA	Grade 4 or 5 by Lown class
Figliozzi	2023	Cohort	104/370	NA	48.52	NA	24.89	39 month	68	Aborted SCD or sustained VT
Chivulescu	2022	Case–control	54/59	NA	NA	NA	NA	NA	NA	Sustained or non‐sustained VT
Fulton	2017	Case–control	5/2	52	57.14	NA	NA	NA	NA	PVCs or VT

### 3.3 Quantitative Analysis of the Effect of CMR LGE on Arrhythmia in MVP Patients

**Figure 2:**
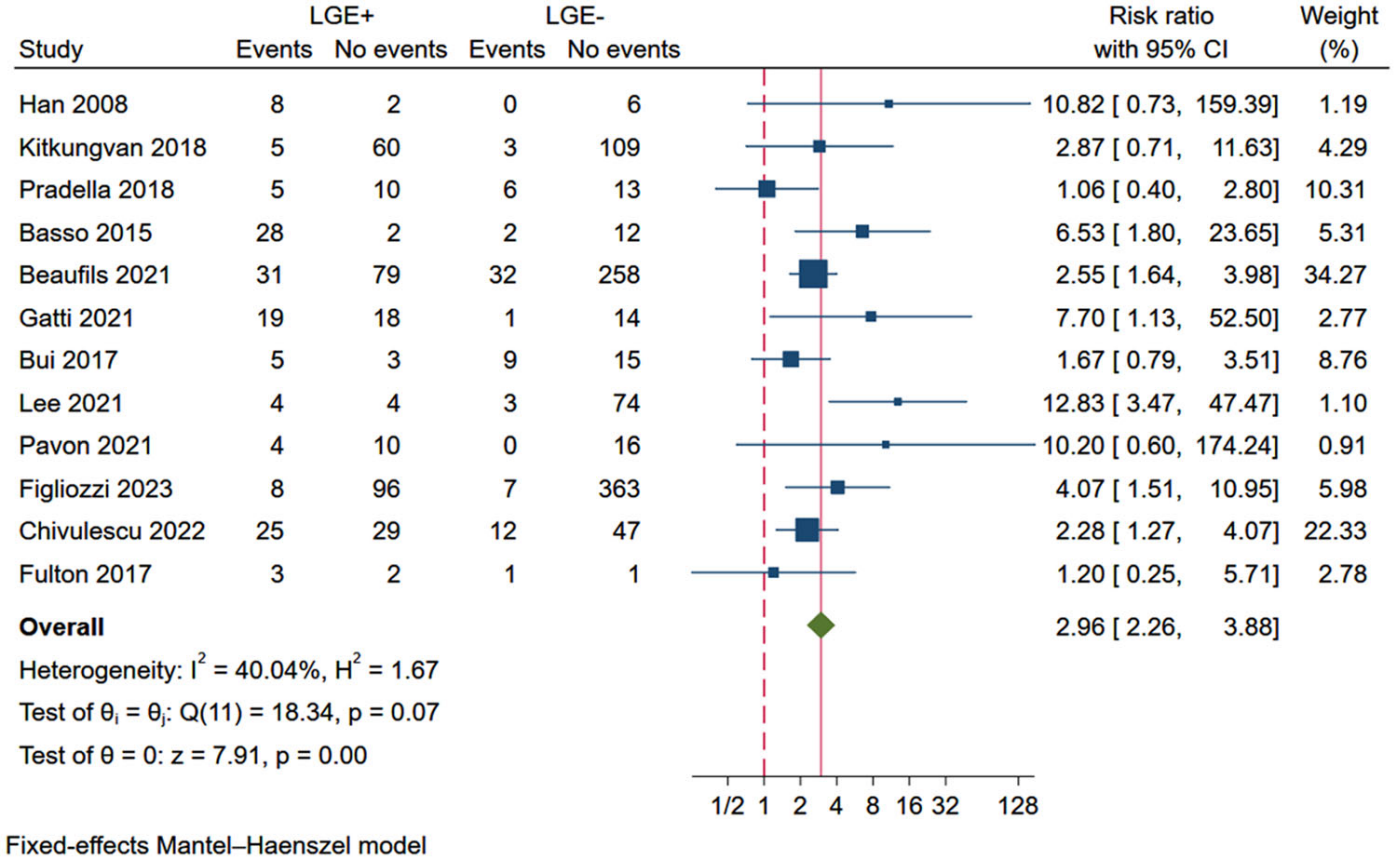
Forest plot showing the RR of VA in MVP patients with LGE+ compared with those with LGE−. CI, confidence interval; LGE, late gadolinium enhancement; RR, relative risk.

Overall, the risk of VA was 2.96 times (95% CI: 2.26−3.88) higher in MVP patients who presented with LGE than in those who did not (Figure 2). However, moderate heterogeneity (*I*² = 40%) was observed. The quality evaluation results are shown in Table S2.

### 3.4 Assessment of the Risk of Publication Bias

**Figure 3:**
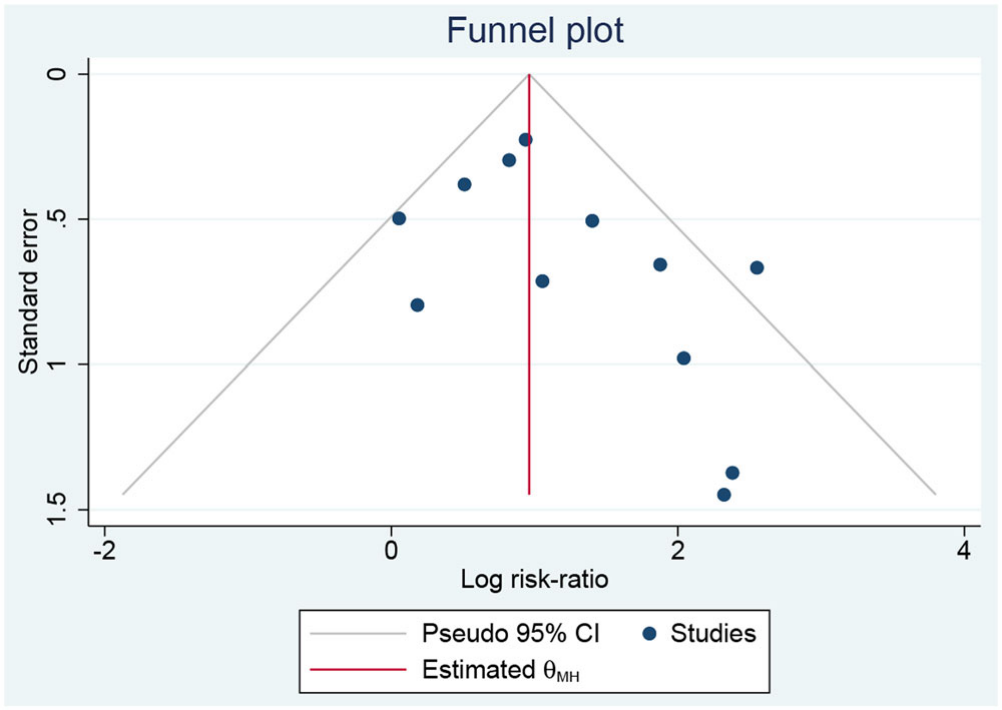
Funnel plot showed publication bias.

In assessing the risk of publication bias, the Egger test yielded a *p* value of 0.099, the Begg test yielded a *p* value of 0.131, and the funnel plot did not show significant asymmetry (Figure 3). These results indicated that there was no significant publication bias in our analysis.

### 3.5 Subgroup Analyses and Meta‐Regression

**Figure 4:**
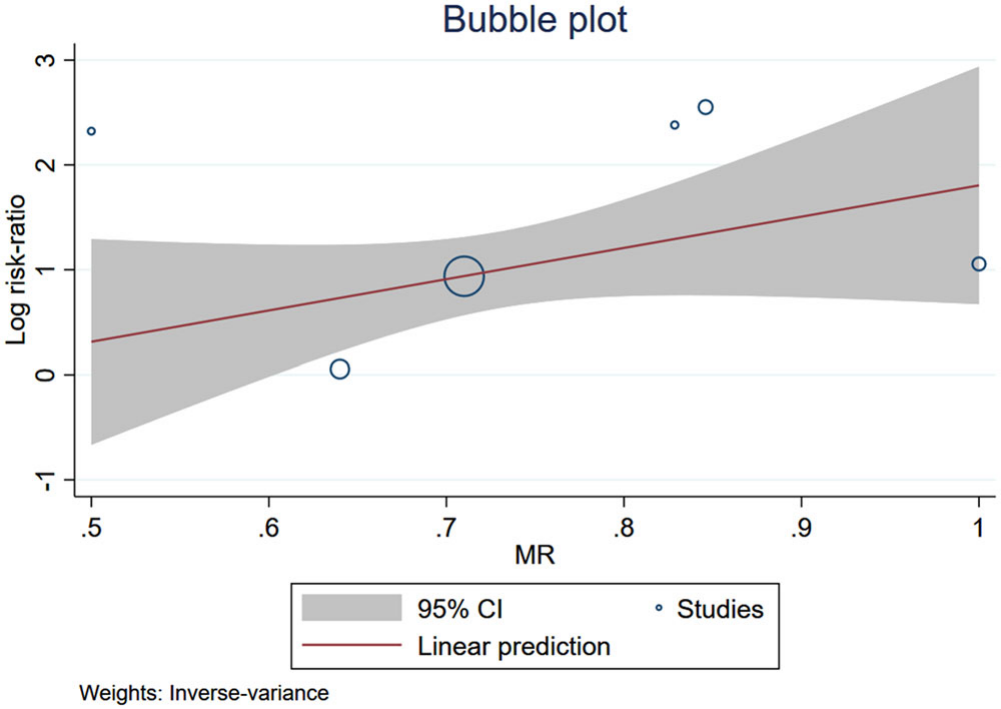
Bubble plots showed meta‐regression analysis. The horizontal coordinate is the proportion of patients with moderate to severe mitral regurgitation, and the vertical coordinate is the RR value associated with LGE and VA.

To examine the factors contributing to heterogeneity, we performed a meta‐regression based on the proportion of moderate and severe MR reported in each study. The *p* value for the chi‐square test described above was 0.079, which did not reach statistical significance (Figure 4). However, only six studies reported MR, which may have led to inaccurate meta‐regression results.

### 3.6 Sensitivity Analyses

Sensitivity analysis revealed that the associations between LGE and VA were consistent, even the two studies with a sample size of less than 20 were excluded (pooled RR 2.91, 95% CI: 2.22−3.83), which confirmed the stability of the study results (Figure S1). In addition, the heterogeneity significantly decreased (*I*² decreased from 40% to 7%) after deleting two studies (Figure S2), and these results were consistent with those of the primary analysis (pooled RR 3.06, 95% CI: 2.29−4.09).

## 4 Discussion

Our meta‐analysis of 12 observational studies and 1464 patients with MVP showed that LGE was associated with a 2.96‐fold increase in the risk of VA (RR 2.96, 95% CI: 2.26−3.88). These findings indicate that there is an association between LGE and the risk of VA among MVP patients. Most of the included studies used case‒control and cross‐sectional designs, with only a small number of cohort studies. The standard of proof was, therefore, considered to be low. However, the findings indicate that LGE tests may be valuable for evaluating VA risk in MVP patients, irrespective of whether the risk is moderate or severe.

Conventional examinations, such as structural parameters measured by ultrasound, are limited in predicting VA risk in MVP patients. It is important to recognize that SCD may occur in MVP patients even without moderate to severe MR and that postmortem findings indicate significant fibrosis in either the left ventricle or the papillary muscle [6].

At the microscopic level, MVP is characterized by a significant proliferation of spongiosa, a glycosaminoglycan, and proteoglycans contained in a spongy elastin net. This causes an interruption of the fibrosa, the underlying, collagen‐rich lower layer located toward the ventricular side of the valve. Secondary effects include fibrotic changes in mitral valve leaflets and narrowing and lengthening of chordae tendineae, which may also be due to the accumulation of glycosaminoglycans and ventricular friction lesions. Some evidence suggests that MVP alone is responsible for papillary and peripapillary fibrosis. Pathological contraction and distortion of the contraction may exert excessive traction on the papillary muscle, thus inducing fibrosis. These fibrotic changes, friction lesions, and myocardial stretching can lead to VAs either through reentry or triggered activity [16].

LGE imaging is a quantitative method for detecting MF and has proven to be valuable in the diagnosis and risk stratification of both ischemic and nonischemic cardiomyopathy [6]. Significant LGE was also observed among MVP patients with complex VA, and several retrospective nonlongitudinal or longitudinal studies have analyzed whether the presence of LGE is associated with VA risk in MVP patients, but the conclusions have been inconsistent. Therefore, a systematic review and pooled analysis of the present findings would be beneficial. Although the included studies had moderate heterogeneity and were all retrospective or cross‐sectional, the primary outcome and sensitivity analysis revealed that LGE was associated with VA risk in patients with MVP. In addition, meta‐regression analysis indicated that the proportions of moderate and severe MR were not significant influencing factors, suggesting that LGE was correlated with VA risk regardless of the presence of moderate or severe MR.

According to the 2022 EHRA expert consensus, arrhythmic MVP is defined according to three criteria: the presence of MVP (with or without mitral annular disjunction); the presence of VA, including frequent (> 5%) or complex VA (NSVT, VT, VF); and no other definite arrhythmic substrates. For MVP patients with aborted SCA or sustained VT, CMR should be performed before ICD for secondary prevention. In addition, CMR is also recommended for MVP patients who experience unexplained syncope or NSVT. Finally, CMR might be useful for risk stratification in MVP patients with other arrhythmic risk factors, including palpitations, T‐wave inversion, polymorphic PVCs, mitral annular disjunction, redundant mitral valve lobes, left atrial enlargement, or reduced LV ejection fraction [17].

MVP is the most common form of cardiac valve disease, but the risk of severe VA and SCD should not be ignored, and effective risk stratification methods are still lacking [6]. The management of MVP focuses primarily on surgical repair or the replacement of the mitral valve to address LV insufficiency resulting from severe MR [18]. However, the management of MVP patients with no or mild MR remains a challenge, and the significance of this study is even more important in this subset of patients. LGE may be indicative of LV wall or papillary muscle fibrosis, which is associated with a higher risk of VA, thus enabling us to distinguish this specific subset of patients from those at genuinely low risk. Such differentiation is clinically important for monitoring MVP patients for VA risk and reducing the risk of sudden death.

Due to the type and quality of the original studies, this meta‐analysis inevitably has the following limitations. First, the absence of prospective cohort studies resulted in prevalent selection bias and confounding bias, and only two studies reported longitudinal rates of SCD. To validate the current conclusions, prospective cohort studies with hard endpoints of arrhythmia events, including persistent VA and SCD, are needed. In addition, only half of the studies reported the proportion of patients with moderate to severe MR, which might have influenced the results of the meta‐regression. Finally, it is not clear whether the quantitative indicators of LGE or the location of its presence are related to VA due to the lack of research data.

## 5 Conclusions

The detection of LGE by CMR could help stratify patients with MVP according to the risk of VA.

## Conflicts of Interest

The authors declare no conflicts of interest.

## Supporting information

Supporting information.

Supporting information.

Supporting information.

Supporting information.

Supporting information.

Supporting information.

## Data Availability

The data supporting this meta‐analysis are from previously reported studies and data sets, which have been cited.
