# Supplementary material for: The Association Between Late Gadolinium Enhancement by Cardiac Magnetic Resonance and Ventricular Arrhythmia in Patients With Mitral Valve Prolapse: A Systematic Review and Meta‐Analysis
Source: Clin Cardiol. 2024 Jul 3;47(7):e24316. doi: 10.1002/clc.24316 (PMC11220671; doi:10.1002/clc.24316)
Supplement: Supplementary file 1 — Supporting information. [file CLC-47-e24316-s001.docx]

Title page

**The association between late gadolinium enhancement by cardiac magnetic resonance and ventricular arrhythmia in patients with mitral valve prolapse: A systematic review and meta-analysis**

Xiaofu Tang^1^; Weiguo Fan, MD^2*^

^1^ The Second affiliated hospital, Jiangxi Medical College, Nanchang University, Nanchang, Jiangxi, China

^2^ Department of Cardiovascular Medicine, The Second affiliated hospital, Jiangxi Medical College, Nanchang University, Nanchang, Jiangxi, China

Xiaofu Tang, E-mail: Liz_0131@163.com

Weiguo Fan, MD, E-mail: fangwg1310@163.com

* Correspondence:
Weiguo Fan, MD

Department of Cardiovascular Medicine, The Second affiliated hospital, Jiangxi Medical College, Nanchang University, Nanchang, Jiangxi, China.

E-mail: fangwg1310@163.com
