# Supplementary material for: The Association Between Late Gadolinium Enhancement by Cardiac Magnetic Resonance and Ventricular Arrhythmia in Patients With Mitral Valve Prolapse: A Systematic Review and Meta‐Analysis
Source: Clin Cardiol. 2024 Jul 3;47(7):e24316. doi: 10.1002/clc.24316 (PMC11220671; doi:10.1002/clc.24316)
Supplement: Supplementary file 2 — Supporting information. [file CLC-47-e24316-s002.docx]

**Table legend:**

**Table 1. Characteristics of studies included in meta-analysis.**

**Figure legends:**

**Figure 1. A flow chart depicting literature screening process.**

**Figure 2. Forest plot showing the RR of VA in MVP patients with LGE+ compared with those with LGE-.** Abbreviations: RR, relative risk; CI, confidence interval; LGE, late gadolinium enhancement.

**Figure 3. Funnel plot showed publication bias.**

**Figure 4. Bubble plots showed meta-regression analysis.** The horizontal coordinate is the proportion of patients with moderate to severe mitral regurgitation, and the vertical coordinate is the RR value associated with LGE and VA.

**Supplementary Table legends:**

**Supplementary Table 1. PubMed, Embase, and Web of Science search string.**

**Supplementary Table 2. The literature quality evaluation of included studies.**

**Supplementary Figure legends:**

**Supplementary Figure 1. Sensitivity analysis showed the pooled result after two studies with sample size of less than 20 were excluded.**

**Supplementary Figure 2. Sensitivity analysis showed the pooled result after two studies with significant impact on heterogeneity were excluded.**
