# Supplementary figures and images for: The Association Between Late Gadolinium Enhancement by Cardiac Magnetic Resonance and Ventricular Arrhythmia in Patients With Mitral Valve Prolapse: A Systematic Review and Meta‐Analysis
Source: Clin Cardiol. 2024 Jul 3;47(7):e24316. doi: 10.1002/clc.24316 (PMC11220671; doi:10.1002/clc.24316)

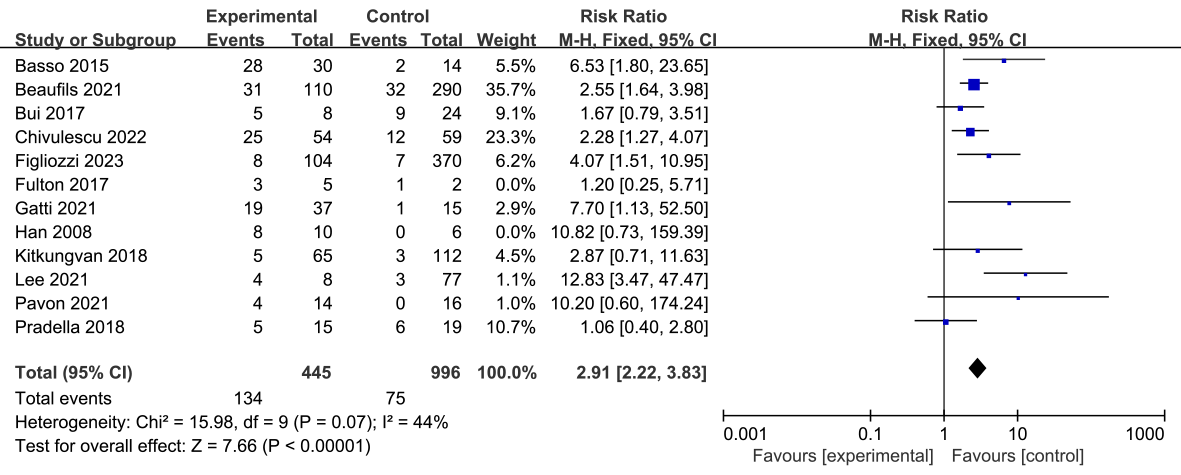

Supplement: Supplementary file 3 — Supporting information. [file CLC-47-e24316-s004.tif]

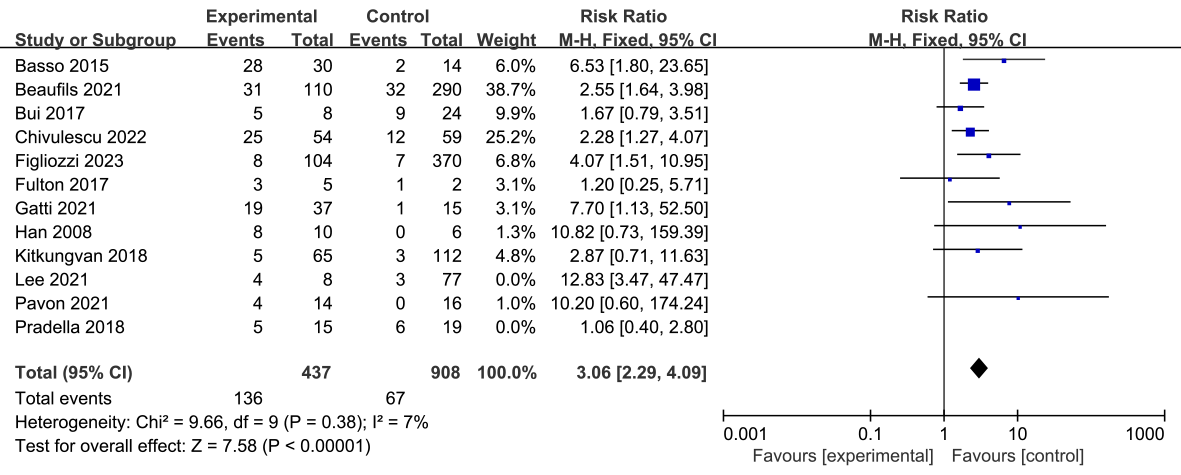

Supplement: Supplementary file 4 — Supporting information. [file CLC-47-e24316-s005.tif]
