# Supplementary material for: The Association Between Late Gadolinium Enhancement by Cardiac Magnetic Resonance and Ventricular Arrhythmia in Patients With Mitral Valve Prolapse: A Systematic Review and Meta‐Analysis
Source: Clin Cardiol. 2024 Jul 3;47(7):e24316. doi: 10.1002/clc.24316 (PMC11220671; doi:10.1002/clc.24316)
Supplement: Supplementary file 5 — Supporting information. [file CLC-47-e24316-s003.docx]

**Supplementary Table 1.** PubMed, Embase, and Web of Science search string.

PubMed

| sequence | search | quantity |
| --- | --- | --- |
| #1 | **Search: Mitral valve prolapse[MeSH Terms]** | **5209** |
| #2 | 1. **Search: Magnetic Resonance Imaging[MeSH Terms]** | **526840** |
| #3 | **Search: Arrhythmias, Cardiac[MeSH Terms]** | **234887** |
| #4 | **Search: Death, Sudden, Cardiac[MeSH Terms]** | **17781** |
| #5 | **Search：(((((((((((((((((((((((((((((Mitral valve prolapse) OR (Mitral Valve Prolapses)) OR (Prolapse, Mitral Valve)) OR (Prolapses, Mitral Valve)) OR (Valve Prolapse, Mitral)) OR (Valve Prolapses, Mitral)) OR (Floppy Mitral Valve)) OR (Floppy Mitral Valves)) OR (Mitral Valve, Floppy)) OR (Mitral Valves, Floppy)) OR (Mitral Click-Murmur Syndrome)) OR (Click-Murmur Syndrome, Mitral)) OR (Mitral Click Murmur Syndrome)) OR (Syndrome, Mitral Click-Murmur)) OR (Systolic Click-Murmur Syndrome)) OR (Click-Murmur Syndrome, Systolic)) OR (Syndrome, Systolic Click-Murmur)) OR (Systolic Click Murmur \Syndrome)) OR (Prolapsed Mitral Valve)) OR (Mitral Valve, Prolapsed)) OR (Mitral Valves, Prolapsed)) OR (Prolapsed Mitral Valves)) OR (Valve, Prolapsed Mitral)) OR (Valves, Prolapsed Mitral)) OR (Click-Murmur Syndrome)) OR (Click Murmur Syndrome)) OR (Click-Murmur Syndromes)) OR (Syndrome, Click-Murmur)) OR (Syndromes, Click-Murmur)) OR (Mitral Valve Prolapse Syndrome)** | **8058** |
| #6 | **Search: LGE** | **3416** |
| #7 | **Search: (((Cardiac magnetic resonance)) OR (CMR)) OR (CMR)** | **69936** |
| #8 | **Search: ((((((((((((((((((((((((((((((((((((((((((MRI)) OR (Magnetic Resonance Imaging)) OR (Imaging, Magnetic Resonance)) OR (NMR Imaging)) OR (Imaging, NMR)) OR (Tomography, NMR)) OR (Tomography, MR)) OR (MR Tomography)) OR (NMR Tomography)) OR (Steady-State Free Precession MRI)) OR (Steady State Free Precession MRI)) OR (Zeugmatography)) OR (Imaging, Chemical Shift)) OR (Chemical Shift Imagings)) OR (Imagings, Chemical Shift)) OR (Shift Imaging, Chemical)) OR (Shift Imagings, Chemical)) OR (Chemical Shift Imaging)) OR (Magnetic Resonance Image)) OR (Image, Magnetic Resonance)) OR (Magnetic Resonance Images)) OR (Resonance Image, Magnetic)) OR (Magnetization Transfer Contrast Imaging)) OR (MRI Scans)) OR (MRI Scan)) OR (Scan, MRI)) OR (Scans, MRI)) OR (Tomography, Proton Spin)) OR (Proton Spin Tomography)) OR (fMRI)) OR (MRI, Functional)) OR (Functional MRI)) OR (Functional MRIs)) OR (MRIs, Functional)) OR (Functional Magnetic Resonance Imaging)) OR (Magnetic Resonance Imaging, Functional)) OR (Spin Echo Imaging)) OR (Echo Imaging, Spin)) OR (Echo Imagings, Spin)) OR (Imaging, Spin Echo)) OR (Imagings, Spin Echo)) OR (Spin Echo Imagings)** | **358856** |
| #9 | **Search: ventricular arrhythmias** | **102425** |
| #10 | **Search: ((((((((((((sudden cardiac death)) OR (sudden cardiac arrest)) OR (Sudden Cardiac Death)) OR (Cardiac Death, Sudden)) OR (Death, Sudden Cardiac)) OR (Cardiac Sudden Death)) OR (Death, Cardiac Sudden)) OR (Sudden Death, Cardiac)) OR (Sudden Cardiac Arrest)) OR (Arrest, Sudden Cardiac)) OR (Cardiac Arrests, Sudden)) OR (Cardiac Arrest, Sudden)** | **41429** |
| #11 | **Search: #5 AND (#6 OR #7 OR #8) AND (#9 OR #10)** | **92** |

Embase

| sequence | search | quantity |
| --- | --- | --- |
| #1 | Search: ‘Mitral valve prolapse’ OR ‘Mitral Valve Prolapses’ OR ‘Prolapse, Mitral Valve’ OR ‘Prolapses, Mitral Valve’ OR ‘Valve Prolapse, Mitral’ OR ‘Valve Prolapses, Mitral’ OR ‘Floppy Mitral Valve’ OR ‘Floppy Mitral Valves’ OR ‘Mitral Valve, Floppy’ OR ‘Mitral Valves, Floppy’ OR ‘Mitral Click-Murmur Syndrome’ OR ‘Click-Murmur Syndrome, Mitral’ OR ‘Mitral Click Murmur Syndrome’ OR ‘Syndrome, Mitral Click-Murmur’ OR ‘Systolic Click-Murmur Syndrome’ OR ‘Click-Murmur Syndrome, Systolic’ OR ‘Syndrome, Systolic Click-Murmur’ OR ‘Systolic Click Murmur Syndrome’ OR ‘Prolapsed Mitral Valve’ OR ‘Mitral Valve, Prolapsed’ OR ‘Mitral Valves, Prolapsed’ OR ‘Prolapsed Mitral Valves’ OR ‘Valve, Prolapsed Mitral’ OR ‘Valves, Prolapsed Mitral’ OR ‘Click-Murmur Syndrome’ OR ‘Click Murmur Syndrome’ OR ‘Click-Murmur Syndromes’ OR ‘Syndrome, Click-Murmur’ OR ‘Syndromes, Click-Murmur’ OR ‘Mitral Valve Prolapse Syndrome’ | 10947 |
| #2 | Search: ‘Cardiac magnetic resonance’ OR ‘CMR’ OR ‘CMR’ OR ‘LGE’ OR ‘MRI’ OR ‘Magnetic Resonance Imaging’ OR ‘Imaging, Magnetic Resonance’ OR ‘NMR Imaging’ OR ‘Imaging, NMR’ OR ‘Tomography, NMR’ OR ‘Tomography, MR’ OR ‘MR Tomography’ OR ‘NMR Tomography’ OR ‘Steady-State Free Precession MRI’ OR ‘Steady State Free Precession MRI’ OR ‘Zeugmatography’ OR ‘Imaging, Chemical Shift’ OR ‘Chemical Shift Imagings’ OR ‘Imagings, Chemical Shift’ OR ‘Shift Imaging, Chemical’ OR ‘Shift Imagings, Chemical’ OR ‘Chemical Shift Imaging’ OR ‘Magnetic Resonance Image’ OR ‘Image, Magnetic Resonance’ OR ‘Magnetic Resonance Images’ OR ‘Resonance Image, Magnetic’ OR ‘Magnetization Transfer Contrast Imaging’ OR ‘MRI Scans’ OR ‘MRI Scan’ OR ‘Scan, MRI’ OR ‘Scans, MRI’ OR ‘Tomography, Proton Spin’ OR ‘Proton Spin Tomography’ OR ‘fMRI’ OR ‘MRI, Functional’ OR ‘Functional MRI’ OR ‘Functional MRIs’ OR ‘MRIs, Functional’ OR ‘Functional Magnetic Resonance Imaging’ OR ‘Magnetic Resonance Imaging, Functional’ OR ‘Spin Echo Imaging’ OR ‘Echo Imaging, Spin’ OR ‘Echo Imagings, Spin’ OR ‘Imaging, Spin Echo’ OR ‘Imagings, Spin Echo’ OR ‘Spin Echo Imagings’ OR ‘late gadolinium enhancement imaging’ | 1236017 |
| #3 | Search: ‘arrhythmias’ OR ‘sudden cardiac death’ OR ‘sudden cardiac arrest’ OR ‘Sudden Cardiac Death’ OR ‘Cardiac Death, Sudden’ OR ‘Death, Sudden Cardiac’ OR ‘Cardiac Sudden Death’ OR ‘Death, Cardiac Sudden’ OR ‘Sudden Death, Cardiac’ OR ‘Sudden Cardiac Arrest’ OR ‘Arrest, Sudden Cardiac’ OR ‘Cardiac Arrests, Sudden’ OR ‘Cardiac Arrest, Sudden’ OR ‘Arrhythmia, Cardiac’ OR ‘Cardiac Dysrhythmia’ OR ‘Dysrhythmia, Cardiac’ OR ‘Cardiac Arrhythmia’ OR ‘Cardiac Arrhythmias’ OR ‘Arrhythmia’ OR ‘Arrythmia’ | 288205 |
| #4 | #1 AND #2 AND #3 | 296 |

Web of science

| sequence | search | quantity |
| --- | --- | --- |
| #1 | Search: (((((((((((((((((((((((((((((TS=(Mitral valve prolapse)) OR TS=(Mitral Valve Prolapses)) OR TS=(Prolapse, Mitral Valve)) OR TS=(Prolapses, Mitral Valve)) OR TS=(Valve Prolapse, Mitral)) OR TS=(Valve Prolapses, Mitral)) OR TS=(Floppy Mitral Valve)) OR TS=(Floppy Mitral Valves)) OR TS=(Mitral Valve, Floppy)) OR TS=(Mitral Valves, Floppy)) OR TS=(Mitral Click-Murmur Syndrome)) OR TS=(Click-Murmur Syndrome, Mitral)) OR TS=(Mitral Click Murmur Syndrome)) OR TS=(Syndrome, Mitral Click-Murmur)) OR TS=(Systolic Click-Murmur Syndrome)) OR TS=(Click-Murmur Syndrome, Systolic)) OR TS=(Syndrome, Systolic Click-Murmur)) OR TS=(Systolic Click Murmur Syndrome)) OR TS=(Prolapsed Mitral Valve)) OR TS=(Mitral Valve, Prolapsed)) OR TS=(Mitral Valves, Prolapsed)) OR TS=(Prolapsed Mitral Valves)) OR TS=(Valve, Prolapsed Mitral)) OR TS=(Valves, Prolapsed Mitral)) OR TS=(Click-Murmur Syndrome)) OR TS=(Click Murmur Syndrome)) OR TS=(Click-Murmur Syndromes)) OR TS=(Syndrome, Click-Murmur)) OR TS=(Syndromes, Click-Murmur)) OR TS=(Mitral Valve Prolapse Syndrome) | 5665 |
| #2 | Search: ((((((((((((((((((((((((((((((((((((((((((((((TS=(Cardiac magnetic resonance)) OR TS=(CMR)) OR TS=(CMR)) OR TS=(LGE)) OR TS=(MRI)) OR TS=(Magnetic Resonance Imaging)) OR TS=(Imaging, Magnetic Resonance)) OR TS=(NMR Imaging)) OR TS=(Imaging, NMR)) OR TS=(Tomography, NMR)) OR TS=(Tomography, MR)) OR TS=(MR Tomography)) OR TS=(NMR Tomography)) OR TS=(Steady-State Free Precession MRI)) OR TS=(Steady State Free Precession MRI)) OR TS=(Zeugmatography)) OR TS=(Imaging, Chemical Shift)) OR TS=(Chemical Shift Imagings)) OR TS=(Imagings, Chemical Shift)) OR TS=(Shift Imaging, Chemical)) OR TS=(Shift Imagings, Chemical)) OR TS=(Chemical Shift Imaging)) OR TS=(Magnetic Resonance Image)) OR TS=(Image, Magnetic Resonance)) OR TS=(Magnetic Resonance Images)) OR TS=(Resonance Image, Magnetic)) OR TS=(Magnetization Transfer Contrast Imaging)) OR TS=(MRI Scans)) OR TS=(MRI Scan)) OR TS=(Scan, MRI)) OR TS=(Scans, MRI)) OR TS=(Tomography, Proton Spin)) OR TS=(Proton Spin Tomography)) OR TS=(fMRI)) OR TS=(MRI, Functional)) OR TS=(Functional MRI)) OR TS=(Functional MRIs)) OR TS=(MRIs, Functional)) OR TS=(Functional Magnetic Resonance Imaging)) OR TS=(Magnetic Resonance Imaging, Functional)) OR TS=(Spin Echo Imaging)) OR TS=(Echo Imaging, Spin)) OR TS=(Echo Imagings, Spin)) OR TS=(Imaging, Spin Echo)) OR TS=(Imagings, Spin Echo)) OR TS=(Spin Echo Imagings)) OR TS=(late gadolinium enhancement imaging) | 607016 |
| #3 | Search: (((((((((((((((((((TS=(arrhythmias)) OR TS=(sudden cardiac death)) OR TS=(sudden cardiac arrest)) OR TS=(Sudden Cardiac Death)) OR TS=(Cardiac Death, Sudden)) OR TS=(Death, Sudden Cardiac)) OR TS=(Cardiac Sudden Death)) OR TS=(Death, Cardiac Sudden)) OR TS=(Sudden Death, Cardiac)) OR TS=(Sudden Cardiac Arrest)) OR TS=(Arrest, Sudden Cardiac)) OR TS=(Cardiac Arrests, Sudden)) OR TS=(Cardiac Arrest, Sudden)) OR TS=(Arrhythmia, Cardiac)) OR TS=(Cardiac Dysrhythmia)) OR TS=(Dysrhythmia, Cardiac)) OR TS=(Cardiac Arrhythmia)) OR TS=(Cardiac Arrhythmias)) OR TS=(Arrhythmia)) OR TS=(Arrythmia) | 120024 |
| #4 | #1 AND #2 AND #3 | 78 |
