# Supplementary material for: The Association Between Late Gadolinium Enhancement by Cardiac Magnetic Resonance and Ventricular Arrhythmia in Patients With Mitral Valve Prolapse: A Systematic Review and Meta‐Analysis
Source: Clin Cardiol. 2024 Jul 3;47(7):e24316. doi: 10.1002/clc.24316 (PMC11220671; doi:10.1002/clc.24316)
Supplement: Supplementary file 6 — Supporting information. [file CLC-47-e24316-s006.docx]

**Supplementary Table 2A.** The literature quality evaluation of case-control studies.

| Author | Year | Study population selection | | | | Comparability between groups | Measurement of exposure factors | | | Total |
| --- | --- | --- | --- | --- | --- | --- | --- | --- | --- | --- |
|  |  | Case determination | Representativeness | Contrast selection | Determination of control |  | Exposure factors | Method | Non-response rate |  |
| Pavon | 2021 | 1 | 1 | 1 | 1 | 1 | 1 | 1 | 1 | 8 |
| Chivulescu | 2022 | 1 | 1 | 1 | 1 | 1 | 1 | 1 | 1 | 8 |
| Fulton | 2017 | 1 | 0 | 1 | 1 | 1 | 1 | 1 | 1 | 7 |

**Supplementary Table 2B.** The literature quality evaluation of cross section studies.

| Author | Year | Research purpose | Population selected | Inclusion and exclusion criteria | Sample characteristics | Data collection tools | Authenticity of the data | Ethical issues | Statistical method | Research results | Value of research | Total |
| --- | --- | --- | --- | --- | --- | --- | --- | --- | --- | --- | --- | --- |
| Han | 2008 | 2 | 2 | 2 | 1 | 2 | 0 | 0 | 2 | 2 | 1 | 14 |
| Kitkungvan | 2018 | 2 | 2 | 1 | 1 | 2 | 0 | 0 | 2 | 2 | 2 | 14 |
| Pradella | 2018 | 2 | 2 | 2 | 1 | 2 | 0 | 0 | 2 | 2 | 2 | 15 |
| Basso | 2015 | 2 | 1 | 1 | 1 | 2 | 0 | 0 | 2 | 2 | 1 | 12 |
| Gatti | 2021 | 2 | 2 | 2 | 1 | 2 | 0 | 0 | 2 | 2 | 1 | 14 |
| Bui | 2017 | 2 | 1 | 1 | 1 | 2 | 0 | 0 | 2 | 2 | 2 | 13 |

**Supplementary Table 2C.** The literature quality evaluation of cohort studies.

| Author | Year | Study population selection | | | | Comparability between groups | Result measurement | | | Total |
| --- | --- | --- | --- | --- | --- | --- | --- | --- | --- | --- |
|  |  | Representativeness | Selection of non-exposed group | Determining exposure factors | Determine outcome indicators |  | Adequately evaluates the results | Follow-up time | Exposed and non-exposed groups were followed |  |
| Beaufils | 2021 | 1 | 1 | 1 | 1 | 1 | 1 | 0 | 1 | 7 |
| Lee | 2021 | 1 | 1 | 1 | 1 | 1 | 1 | 0 | 1 | 7 |
| Figliozzi | 2023 | 1 | 1 | 1 | 1 | 2 | 1 | 1 | 1 | 9 |
